# Cognitive functioning and functional brain networks in postoperative WHO grade I meningioma patients

**DOI:** 10.1007/s11060-018-2987-1

**Published:** 2018-09-15

**Authors:** David van Nieuwenhuizen, Linda Douw, Martin Klein, Saskia M. Peerdeman, Jan J. Heimans, Jaap C. Reijneveld, Cornelis J. Stam, Arjan Hillebrand

**Affiliations:** 1 Department of Neurology, Amsterdam University Medical Centers, Location VUmc, Amsterdam, The Netherlands; 2 Department of Medical Psychology, Amsterdam University Medical Centers, Location VUmc, Amsterdam, The Netherlands; 3 Department of Clinical Neurophysiology and MEG Center, Amsterdam University Medical Centers, Location VUmc, Amsterdam, The Netherlands; 4 Department of Anatomy and Neurosciences, Amsterdam University Medical Centers, Location VUmc, Amsterdam, The Netherlands; 5 Department of Neurosurgery, Amsterdam University Medical Centers, Location VUmc, Amsterdam, The Netherlands; 6 Brain Tumor Center Amsterdam, Amsterdam University Medical Centers, Location VUmc, Amsterdam, The Netherlands; 7 Department of Neurology, Amphia Hospital, Molengracht 21, 4818 CK Breda, The Netherlands

**Keywords:** Cognitive functioning, Functional connectivity, Magnetoencephalography, Resting-state networks, Meningioma, Minimum spanning tree

## Abstract

**Introduction:**

Meningioma patients often have subtle cognitive deficits that might be attributed to the tumor itself, to surgical treatment, or to the occurrence of seizures and their treatment. Magnetoencephalography (MEG) analysis of resting-state functional networks (RSNs) could help to understand the neurophysiological basis of cognitive impairment in these patients. We explored the correlation between RSN functional connectivity and topology of functional networks on the one hand, and cognition on the other hand in WHO grade I meningioma patients.

**Methods:**

Twenty adult WHO grade I meningioma patients who had undergone tumor resection, as well as 20 healthy matched controls, were included. Neuropsychological assessment was done through a standardized test battery. MEG data were recorded, and projected to the anatomical space of the Automated Anatomical Labeling atlas. Functional connectivity (PLI), within the default mode network (DMN) and the bilateral frontoparietal networks were correlated to cognitive performance. Minimum spanning tree (MST) characteristics were correlated with cognitive functioning.

**Results:**

Compared to healthy controls, meningioma patients had lower working memory capacity (*p* = 0.037). Within the patient group, lower working memory performance was associated with lower DMN connectivity and a lower maximum MST degree in the theta band (resp. *p* = 0.044 and *p* = 0.003).

**Conclusions:**

This study shows that cognitive functioning is correlated with functional connectivity in the default mode network and hub-pathology in WHO grade I meningioma patients. Future longitudinal studies are needed to corroborate these findings and to further investigate the pathophysiology of cognitive deficits and possible changes in functional brain networks in meningioma patients.

**Electronic supplementary material:**

The online version of this article (10.1007/s11060-018-2987-1) contains supplementary material, which is available to authorized users.

## Introduction

Meningiomas are the most frequently diagnosed primary CNS tumors. Ninety percent of meningiomas are WHO grade I tumors, and have a relatively benign disease course [1]. Notwithstanding this favorable prognosis, many WHO grade I meningioma patients show subtle cognitive impairments that affect health-related quality of life [2]. Cognitive impairments may be due to the tumor, peritumoral edema, surgical treatment, or the occurrence of seizures and their treatment with anti-epileptic drugs.

Cognitive deficits in brain tumor patients tend to be global and cannot be explained unequivocally by local damage alone [3]. It has become clear in recent years that the brain is a complex network of interacting brain regions, and that an optimal organization of functional brain networks is required for adequate cognitive functioning [4, 5]. Brain tumors lead to global alterations in functional interactions, even between brain regions remote from the tumor [6–8]. This insight has increased our understanding of the symptoms in these patients: the severity of cognitive symptoms may be explained by alterations of specific networks, possibly depending on pathophysiology and growth pattern [9].

Functional brain networks can be reconstructed using magnetoencephalography (MEG), electroencephalography (EEG) or functional magnetic resonance imaging (fMRI). Using MEG, it has been shown that glioma patients have globally disturbed brain network organization during the resting-state [3, 6–12]. Increased whole-brain functional connectivity in the delta, theta and gamma frequency bands has been associated with poorer cognitive performance in glioma patients, and global network properties such as clustering and path length also relate to patients’ cognitive status [10–12]. However, meningiomas differ from gliomas in many aspects: meningiomas are extra-axial, usually slow growing tumors, whereas gliomas are infiltrative, and very often fast growing intra-axial tumors. It is as yet unclear if and how functional networks are affected by meningiomas. Previous studies have shown that high cost/high value hubs of human brain networks are more likely to be anatomically abnormal than non-hubs in many (if not all) brain disorders, suggesting that hub-damage may be associated with clinically significant cognitive impairments [13].

Studies have shown that the functional brain network is composed of several sub-networks, known as resting-state networks (RSNs), and these RSNs have been linked to cognitive functioning [14, 15]. The default mode network (DMN), which is mostly deactivated during tasks, is the most consistently described RSN, and its resting-state activity has been related to performance in several cognitive domains, including attention and working memory, in both health and disease [16]. Left-sided and right-sided frontoparietal networks (FPN) are crucial for attention, language and memory processing [15]. Correlations between decreased RSN activity and cognitive deficits have been described in several neurological disorders, such as dementia, stroke, and epilepsy [16–18]. In the majority of studies that have investigated RSNs and their associations with cognition, fMRI has been used. MEG provides a direct measure of neuronal activity with high temporal resolution, making it an excellent non-invasive technique for the characterization of functional brain networks [19]. The RSNs that are typically observed in fMRI have been replicated in MEG [20].

Rather than focusing on specific sub-networks, the topology of the functional network as a whole can also be characterized using graph theoretical tools. Although conventional graph theoretical analyses are helpful in understanding disease mechanisms [4, 5], there are methodological difficulties that may hamper interpretation of results [21]; graph measures are influenced by the size of the network (i.e. the number of nodes), network sparsity (percentage links present), and the average degree (i.e. the number of connections per node). The minimum spanning tree (MST), on the contrary, is a unique reconstruction of the backbone of the functional network, provided that all the weights are unique [22]. Recent studies have shown that MST network analyses are able to detect network changes within patient groups (e.g. [23, 24]).

Here, we explored the correlations between band-specific connectivity within three MEG RSNs and the MST on the one hand, and cognitive functioning on the other hand in WHO grade I meningioma patients. We hypothesized that lower network connectivity in meningioma WHO grade I patients is related to poorer cognitive performance. Furthermore, we investigated whether we could detect correlations between the topology of functional networks and cognitive functioning.

## Methods

### Patients

Consecutive adult WHO grade I meningioma patients who had undergone tumor resection, without adjuvant treatment, were assessed.

Excluded from the study were patients with atypical or malignant meningioma (WHO grade II or III), as well as patients who suffered from one of the following medical conditions that may interfere with normal cognitive functioning: other central nervous system (CNS) or non-CNS malignancy, cerebrovascular pathology, congenital CNS malformations, multiple sclerosis, Parkinson’s disease, organic psychosis, dementia, or schizophrenia. Furthermore, patients had to have sufficient proficiency of the Dutch language to be able to carry out the cognitive tests.

### Healthy controls

Healthy controls providing normative data for cognitive functioning were selected from the Maastricht Aging Study, which comprises a cross-sectional study into the biomedical and psychological determinants of cognitive aging of 2000 healthy individuals aged 24–81 years [25]. Patients and healthy controls were individually matched with respect to sex, age, and educational level. Educational level was assessed by a Dutch scoring system consisting of an eight-point scale, ranging from unfinished primary education (level 1) to university level (level 8) [26].

### Ethics statement

Ethical approval was granted by the VU University Medical Ethics Committee. All patients gave written informed consent before participating. All clinical investigations were conducted according to the Declaration of Helsinki.

### Neuropsychological assessment

A wide range of cognitive functions was assessed by means of a standardized test battery (Appendix 1). The total time required to complete the battery was approximately 60 min. Individual cognitive test scores were converted into z-scores using the means and standard deviations (SDs) of the scores for the age-, sex-, and education-matched healthy controls as a reference. Subsequently, z-scores were transformed into the following six cognitive domains: executive functioning, working memory, verbal memory, attention, information processing speed, and psychomotor speed [27]. Cognitive dysfunction was defined as a z-score ≥ 1.5 SD below the mean for the healthy controls.

### Magnetoencephalography (MEG)

Postoperative MEG recordings were made in a magnetically shielded room (VacuumSchmelze GmbH, Hanua, Germany) using a 151-channel whole-head MEG system (CTF Systems Inc., Port Coquitlam, BC, Canada). Approximately 5 min of MEG data were recorded with a third-order software gradient, a sample frequency of 625 Hz and a passband of 0–100 Hz.

At the start and end of the measurement, the head position relative to the coordinate system of the helmet was determined by leading small currents through three head-position coils situated at the left and right pre-auricular points and the nasion. Changes in head position smaller than 0.5 cm during the recording were accepted. The MEG recordings were performed in a no task, eyes-closed and eyes-open resting state condition, followed by the delayed non-matching-to-sample (DNMTS) task [28]. Only data from the eyes-closed condition were analysed here. For each participant, the continuous approximately 5 min long resting-state, eyes-closed recording was divided into epochs of 6.555 s. Channels and epochs were visually inspected for representing an alert eyes-closed state. Epochs or channels were rejected when containing system related or physiological artefacts or too much environmental noise, leading to discarding of on average 6.3 channels (range 3–10). On average 35.3 epochs (range 25–47) were selected and subsequently projected to source-space (see below).

### Anatomical MRI

Postoperative structural magnetic resonance images (MRIs) were used for co-registration [29], and the outline of the scalp as obtained from the co-registered MRI was used as a multi-sphere headmodel [30]. The co-registered MRI was then spatially normalized to a template MRI using the SEG toolbox in SPM8. The Automated Anatomical Labeling (AAL) atlas was used to label the voxels in a subject’s normalized co-registered MRI. Subcortical structures were removed, and the voxels in the remaining 78 cortical regions of interest (ROIs) were used for further analyses, after inverse transformation to the patient’s co-registered MRI.

### Time series estimation for regions-of-interest

We used the beamformer approach [29] as described by Hillebrand et al. [31], which reconstructs a single time series of neuronal activity for each ROI and frequency band separately. For the beamformer computations we used the data covariance for, on average, 231 s (range 164–308 s) of data, and used a unity matrix for the noise covariance. Three frequency bands were analyzed: theta (4–8 Hz), lower alpha (8–10 Hz), and upper alpha (10–13 Hz). Five artefact free epochs of 4096 samples (6.555 s) were subsequently selected [DvN] and analyzed using Brainwave v0.9.58 [authored by C.S.; available at http://home.kpn.nl/stam7883/brainwave.html].

### Functional connectivity

The phase lag index (PLI) was used to assess functional connectivity between the reconstructed source-space signals. The PLI is a measure that is relatively insensitive to the effects of volume conduction and field spread [32]. For each subject, the PLI was calculated for all possible ROI pairs. The functional connectivity within one of the predefined RSNs described below was then computed by averaging all PLI values between the ROIs within that sub-network.

### Resting-state networks

We defined MEG RSNs by selecting only those connections between the ROIs within literature-based RSNs [15]. We selected three RSNs that are of interest for cognitive functioning in the domains that are frequently affected in meningioma patients, namely the default mode network (DMN), and the left and the right frontoparietal network (FPN) (see Table S1 for included ROIs) [2]. Only if the meningioma patients differed from healthy controls regarding cognitive functioning, we investigated associations between the significantly different cognitive domain(s) and average functional connectivity within these three RSNs.

### Minimum spanning tree analyses

The minimum spanning tree of an undirected weighted graph is a unique subgraph that connects all the nodes in such a way that the total cost (the sum of all the edge distances) is minimized without forming cycles. For each subject, each epoch, and each frequency band separately, we constructed the MST based on the PLI adjacency matrix by applying Kruskal’s algorithm [23, 33]. Subsequently we characterized the topology of the MST using the following global metrics: maximum degree, leaf fraction, mean eccentricity, maximum betweenness centrality (BC), and tree hierarchy (T_H_) [23, 33, 34]. These metrics were averaged over epochs for each subject and frequency bands.

### Statistics

Because of the non-normal distribution of the data and small sample size, Mann–Whitney *U* tests were used to test for differences in cognitive functioning between patients and healthy controls. Furthermore, Kendall’s Tau coefficients (two-tailed) were used to determine the associations between cognitive functioning and the mean PLI within each RSN and global MST metrics. In the statistical analyses performed using SPSS (version 20.0 for Windows) statistical significance was set at *p* < 0.05.

## Results

### Sociodemographic and clinical characteristics

Of the 38 patients who met the inclusion criteria, 18 patients were excluded; of these 18, 10 declined participation as they expected testing to be too burdensome. In the remaining 8 patients, either MRI or MEG could not be used for analysis due to artefacts. No significant differences for age, sex, and educational level were found between the included and excluded patients. Mean time between resection and testing (MEG and neuropsychological assessment) was 1.7 years (range 0.4–4.6 years). As a result of the matching procedure, the 20 remaining patients and healthy controls did not differ in age, sex, or educational level (Table 1).

**Table 1:** Patients’ sociodemographic and clinical characteristics

	Meningioma patients (*n* = 20)	Healthy controls (n = 20)	
	Mean	Mean	P*
Characteristics			
Mean age in years^a^	51.4 (10.9)	51.6 (11.0)	0.99
Sex, # males (%)	6 (30.0)	6 (30.0)	
Educational level (median and range)^b^	4 (6)	4 (6)	1.0
Premorbid intelligence			
Dutch adult reading test (raw scores)	88.3 (11.3)	n/a	
Time between resection and testing (years*)	1.7 (0.4–4.6)	n/a	
Functional/performance status			
Barthel	19.8 (0.8)	n/a	
Location of tumor (#)			
Frontal	12		
Parietal	4		
Temporal	2		
Occipital	2		

Data are mean and standard deviation (given between brackets), unless indicated otherwise

n/a Not applicable or not available; # number

*Mean years (and range) since tumor resection and neurocognitive testing

^a^Non-parametric *t* test

### Cognitive functioning

Patients had significantly lower working memory capacity (Mann–Whitney U = 268.000, *p* = 0.037) compared to healthy controls. No statistically significant differences were found between patients and healthy controls in executive functioning, verbal memory, attention functioning, information processing speed, or psychomotor speed (Table 2).

**Table 2:** Cognitive functioning in meningioma patients compared to healthy controls

Cognitive domain	Meningioma patients N = 20	Healthy controls N = 20	P*	Mann–Whitney U
Executive functioning	− 0.0960 (1.011)	0.0312 (0.760)	0.477	178.000
Psychomotor speed	− 0.2921 (0.981)	0.0243 (0.730)	0.159	237.000
Working memory	− 0.5348 (1.004)	0.0127 (0.8970)	**0.037**	268.000
Information processing	− 0.3352 (1.130)	0.0176 (0.992)	0.467	234.500
Attention	− 0.2682 (1.162)	0.0122 (0.814)	0.347	184.000
Verbal memory	0.1466 (1.050)	0.0715 (0.610)	0.224	163.000

Results are mean with (standard deviation)

For the neuropsychological characteristics Z-scores derived from the mean and SD of the healthy controls are displayed

*Significantly different (*p* < 0.05) from the Meningioma patients using Mann–Whitney U-test (one-tailed)

### Associations between functional connectivity and working memory

Correlational analyses (Kendall’s tau) showed significant positive associations between theta band DMN connectivity and working memory (r = 0.326; p = 0.044). No significant associations were found between working memory capacity and connectivity in the other RSNs, nor for other frequency bands. Furthermore, working memory z-scores also correlated positively with the MST maximum degree in the theta band (r = 0.503; p = 0.003). No significant associations were found between working memory scores and other MST metrics, nor for other frequency bands (Table 3). Post hoc analyses showed no significant associations between average functional connectivity of the global network and cognitive functioning (Table 4).

**Table 3:** Correlations between working memory and functional connectivity

	PLI DMN Theta	PLI FPL Theta	PLI FPR Theta	MST degree Theta	MST leaf Theta	MST eccentricity Theta	MST BC Theta	MST T_H_ Theta
Working memory	**r** = **0.326**^a^ ***p*** = **0.044**	r = 0.221^a^ *p* = 0.173	r = − 0.168^a^ *p* = .299	**r** = **0.503**^a^ ***p*** = **0.003**	r = 0.117^a^ *p* = 0.474	r = − 0.184^a^ *p* = 0.267	r = 0.174^a^ *p* = 0.284	r = 0.084^a^ *p* = 0.604

	PLI DMN Lower alpha	PLI FPL Lower alpha	PLI FPR Lower alpha	MST degree Lower alpha	MST leaf Lower alpha	MST eccentricity Lower alpha	MST BC Lower alpha	MST T_H_ Lower alpha
Working memory	r = − 0.005^a^ *p* = 0.974	r = 0.174^a^ *p* = 0.270	r = 0.126^a^ *p* = 0.436	r = − 0.065^a^ *p* = 0.695	r = 0.085^a^ *p* = 0.603	r = − .048^a^ *p* = 0.770	r = 0.274^a^ *p* = 0.092	r = 0.084^a^ *p* = 0.604

	PLI DMN Upper alpha	PLI FPL Upper alpha	PLI FPR Upper alpha	MST degree Upper alpha	MST leaf Upper alpha	MST eccentricity Upper alpha	MST BC Upper alpha	MST T_H_ Upper alpha
Working memory	r = 0.063^a^ *p* = 0.697	r = 0.211^a^ *p* = 0.194	r = 0.189^a^ *p* = 0.243	r = 0.123^a^ *p* = 0.454	r = 0.069^a^ *p* = 0.673	r = 0.076^a^ *p* = 0.647	r = − .090^a^ *p* = 0.581	r = 0.032^a^ *p* = 0.845

*PLI* Phase Lag Index, *DMN* default mode network, *FPL* fronto parietal network left, *FPR* fronto parietal network right, *MST* minimum spanning tree, *BC* betweenness centrality, *T*_*H*_ hierarchy

Correlation is significant at the *p* < 0.05 level

^a^Nonparametric correlation, Kendall’s tau_b test, r = correlation coefficient

**Table 4:** Correlations between cognitive functioning and functional connectivity of the global network

Cognitive domain	PLI mean Theta band	PLI mean Lower alpha band	PLI mean Upper alpha band
Executive functioning	r = − 0.330^a^ *p* = 0.058	r = − 0.281^a^ *p* = 0.103	r = − 0.131^a^ *p* = 0.448
Psychomotor speed	r = − 0.037^a^ *p* = 0.820	r = − 0.242^a^ *p* = 0.136	r = − 0.053^a^ *p* = 0.745
Working memory	r = − 0.005^a^ *p* = 0.974	r = 0.126^a^ *p* = 0.436	r = − 0.063^a^ *p* = 0.697
Information processing	r = − .0.106^a^ *p* = 0.516	r = − 0.195^a^ *p* = 0.230	r = .0.016^a^ *p* = 0.922
Attention	r = − 0.040^a^ *p* = 0.820	r = − 0.124^a^ *p* = 0.472	r = − 0.236^a^ *p* = 0.172
Verbal memory	r = − 0.177^a^ *p* = 0.293	r = − 0.310^a^ *p* = 0.064	r = − 0.171^a^ *p* = 0.310

*PLI* Phase Lag Index

Correlation is significant at the *p* < 0.05 level

^a^Nonparametric correlation, Kendall’s tau_b test, r = correlation coefficient

## Discussion

In this MEG study we found that postoperative WHO grade I meningioma patients had cognitive deficits in the working memory domain. This decrease in working memory performance was associated with lower functional connectivity in the DMN and lower MST maximum degree in the theta band.

Previous studies have shown that pre- and postoperative meningioma patients have cognitive deficits, including in the working memory domain, in concordance with our current findings [2].

Prior to the present study, no studies had been performed on functional connectivity in WHO grade I meningioma patients. In previous studies on patients with other types of brain tumors (gliomas), associations between functional connectivity and cognitive functioning were found mainly in the theta, lower and upper alpha bands [8–10]. Other studies have revealed associations between theta band connectivity on the one hand and working memory and attention on the other hand in healthy subjects. The theta band may be especially important for working memory [35]. Therefore we focused on these frequency bands in the current work.

The DMN has mainly been described in fMRI studies as a network that is deactivated during task performance, while its resting-state level of connectivity correlates with attention and working memory performance. Decreased connectivity of the DMN has been associated with deficits in attentional control and working memory, and our results corroborate these findings [16].

In the present study, post-hoc analyses did not show significant associations between average functional connectivity of the global network and cognitive functioning. This is somewhat different from findings in previous studies in glioma patients [9–11, 36]. Bosma et al. found a large number of significant, both positive and negative, correlations between short-distance and interhemispheric functional connectivity (PLI) and cognitive performance in low-grade glioma patients [10]. Hu et al. reported positive correlations between cognition and “small world” characteristics in the alpha and high gamma band in glioma patients [36]. It is not known whether these conflicting results are due to the differences in methodology (source space versus sensor space; average functional connectivity versus RSN connectivity) or due to differences between tumor types.

Previous work has revealed that global alterations in connectivity can occur in the presence of a lesion, including in regions in the contralateral hemisphere [7, 37]. Moreover, the impact of lesions in an anatomically realistic model of the human brain relies to a large extent on the importance, or ‘hub’ status, of the lesioned area in the anatomical network [37, 38]. A previous fMRI study on DMN connectivity in glioma patients showed significant differences in DMN connectivity when comparing tumors located within the anterior cingulate cortex and the left lateral parietal cortex, suggesting that the location of the tumor is important for the extent to which the DMN connectivity is affected [39]. However, post-hoc analyses of our data did not reveal significant differences in cognition or RSN connectivity between the patients with frontal (60%, see Table S1) and non-frontal meningiomas (40%). An explanation for the difference between our results and those by Harris and colleagues could be that intra-axial, infiltratively growing gliomas might influence DMN connectivity in a different way than extra-axial located meningiomas [11].

Prior to the present study, no studies had been performed on MST in WHO grade I meningioma patients. MST analysis captures important information about the topology of the full network [40]. Previous MEG studies in patients with Parkinson’s disease and in patients with multiple sclerosis using MST demonstrated changes in MST metrics in the alpha 1 and alpha 2 band which were associated with poorer cognitive performance [23, 24]. The conclusions from these studies are that lower cognitive performance related to a more line-like (“less integrated”) network configuration. The MST findings of the present study are therefore loosely consistent with these previous results.

A number of limitations of this study should be mentioned. First of all, a causal relation of the association between functional connectivity of the DMN and topology of functional networks on the one hand and working memory on the other hand, remains unclear because of the cross-sectional nature of this study and the lack of MEG recordings in a control group. The aim of our study, however, was not to assess causality nor to compare meningioma patients and healthy controls, but to investigate the correlation between functional RSN connectivity and topology of functional networks on the one hand and cognitive functioning on the other hand within a group of WHO grade I meningioma patients. Secondly, as part of our analysis approach we normalized the patients’ MRIs to a template brain (and then labelled voxels using a standard (AAL) atlas), while our patients had altered anatomy due to the tumor, and this discrepancy might have influenced our results. Thirdly, since a preoperative cognitive assessment and MEG recordings were lacking, differentiation between tumor and treatment related factors and their effect on cognition, functional connectivity and functional network topology could not be established, hampering the understanding of the pathophysiology of cognitive deficits and possible changes in functional connectivity and functional network topology in meningioma patients during the disease course. Finally, due to the small number of patients in this study, we did not apply statistical corrections for the number of tests (one for each resting-state network and MST measure the different frequency band). For the same reason, the role of tumor location on cognitive functioning was not studied. Our study is the first, exploratory study on functional connectivity in WHO grade I meningioma patients, and corrections for multiple testing would have reduced power and increased Type II errors, thereby hindering advances in the field. We focused on interpreting general patterns of the findings instead, but we report exact P-values of all individual statistical comparisons for ease of survey.

In conclusion, significant correlations were found between working memory deficits and decreased functional connectivity in the default-mode network, and with hub-pathology. Future studies are needed to further investigate the pathophysiology of cognitive deficits and possible changes in functional connectivity and function network topology in meningioma patients during the disease course and due to treatment. Better understanding of the pathophysiology of cognitive deficits may be helpful in determining the clinical value and optimal timing of treatment of meningioma patients.

### Electronic supplementary material

Below is the link to the electronic supplementary material.

Supplementary material 1 (DOCX 14 KB)

## Appendix 1: Description of the neuropsychological test battery

### Test domains

#### Overall cognitive performance Intelligence

##### The Dutch Adult Reading Test (DART) [41]

The Dutch version of the New Adult Reading Test provides a measure of premorbid capacity based on verbal ability.

#### Perception

##### Line Bisection Test [42]

This test is a device for measuring unilateral neglect, which is usually a sequel of massive right hemisphere lesions. Noticeable errors are most often made by patients with visual field defects who tend to underestimate the side of the line opposite to the defective field. Outcome measures are horizontal and vertical deviations.

#### Memory

##### Auditory Verbal Learning Test (AVLT) [43]

This version of the Rey Auditory Verbal Learning Test calls for various aspects of verbal learning and recall. Measures used for analysis are: memory performance on trial 1 as indicator of immediate recall, total recall after five trials, delayed recall and recognition after 20 min as indicators of memory consolidation into long-term memory, and a delta score as a measure of learning capacity.

##### Working Memory Task (WMT) [44]

This task is designed to measure the speed of memory processes. The underlying principle is that the extra time needed to complete a test in which there is a stepwise increase in the amount of information to be kept in memory, is a measure of the ease at which information is processed in working memory. Capacity is measured by using the slope and intercept as a function of the number of letters to be kept in working memory.

#### Attention and executive function

##### Stroop Color-Word Test (SCWT) [43]

This test is a selective attention task aiming at measuring interference susceptibility and consists of three subtasks with increasing task complexity.

##### Categoric Word Fluency [45]

This is a task requiring the generation of words from specific semantic categories (animals) within a limited time.

##### Concept Shifting Test (CST) [46]

This test, which has two conditions of complexity, predominantly measures functions associated with executive function, especially visual scanning and conceptual tracking. The motor component of this task is measured by three dummy conditions in which no neurocognitive capacity except for graphomotor speed is required.

#### Speed

##### Letter–Digit Substitutiuon Test (LDST) [47]

This test, measures psychomotor speed that is relatively unaffected by a decline in intellectual ability. Participants are required to replace the randomized letters with the appropriate digit indicated by the key, as quickly as possible.

## Abbreviations

RSN: Resting-state network
PLI: Phase lag index
DMN: Default mode network
FPN: Frontoparietal network
MEG: Magnetoencephalography
EEG: Electroencephalography
fMRI: Functional magnetic resonance imaging
BOLD: Blood-oxygenation-level-dependent
CNS: Central nervous system
AAL: Automated Anatomical Labeling
MST: Minimum spanning tree
