# Supplementary material for: Cognitive functioning and functional brain networks in postoperative WHO grade I meningioma patients
Source: J Neurooncol. 2018 Sep 15;140(3):605–13. doi: 10.1007/s11060-018-2987-1 (PMC6267232; doi:10.1007/s11060-018-2987-1)
Supplement: Supplementary file 1 — Supplementary material 1 (DOCX 14 KB) [file 11060_2018_2987_MOESM1_ESM.docx]

**Table S1:**Additional patient characteristics: lateralization and localization of the resected meningioma

| **Patient** | **Age** | **Gender** | **Lateralization** | **Localization** |
| --- | --- | --- | --- | --- |
| 1 | 50 | male | left | frontal |
| 2 | 53 | male | left | parietal |
| 3 | 37 | female | left | frontal |
| 4 | 35 | female | right | temporal |
| 5 | 45 | female | left | frontal |
| 6 | 31 | female | left | temporal |
| 7 | 59 | female | left | frontal |
| 8 | 68 | female | left | parietal |
| 9 | 46 | female | right | parietal |
| 10 | 47 | female | both | frontal |
| 11 | 48 | female | left | frontal |
| 12 | 54 | female | left | occipital |
| 13 | 63 | female | right | frontal |
| 14 | 42 | female | both | frontal |
| 15 | 55 | male | right | occipital |
| 16 | 67 | male | right | frontal |
| 17 | 46 | male | both | frontal |
| 18 | 67 | female | right | parietal |
| 19 | 64 | male | left | frontal |
| 20 | 51 | female | both | frontal |
